# *Bacillus thuringiensis* Cry34Ab1/Cry35Ab1 Interactions with Western Corn Rootworm Midgut Membrane Binding Sites

**DOI:** 10.1371/journal.pone.0053079

**Published:** 2013-01-04

**Authors:** Huarong Li, Monica Olson, Gaofeng Lin, Timothy Hey, Sek Yee Tan, Kenneth E. Narva

**Affiliations:** Dow AgroSciences LLC, Indianapolis, Indiana, United States of America; Ghent University, Belgium

## Abstract

**Background:**

*Bacillus thuringiensis* (Bt) Cry34Ab1/Cry35Ab1 are binary insecticidal proteins that are co-expressed in transgenic corn hybrids for control of western corn rootworm, *Diabrotica virgifera virgifera* LeConte. Bt crystal (Cry) proteins with limited potential for field-relevant cross-resistance are used in combination, along with non-transgenic corn refuges, as a strategy to delay development of resistant rootworm populations. Differences in insect midgut membrane binding site interactions are one line of evidence that Bt protein mechanisms of action differ and that the probability of receptor-mediated cross-resistance is low.

**Methodology/Principal Findings:**

Binding site interactions were investigated between Cry34Ab1/Cry35Ab1 and coleopteran active insecticidal proteins Cry3Aa, Cry6Aa, and Cry8Ba on western corn rootworm midgut brush border membrane vesicles (BBMV). Competitive binding of radio-labeled proteins to western corn rootworm BBMV was used as a measure of shared binding sites. Our work shows that ^125^I-Cry35Ab1 binds to rootworm BBMV, Cry34Ab1 enhances ^125^I-Cry35Ab1 specific binding, and that ^125^I-Cry35Ab1 with or without unlabeled Cry34Ab1 does not share binding sites with Cry3Aa, Cry6Aa, or Cry8Ba. Two primary lines of evidence presented here support the lack of shared binding sites between Cry34Ab1/Cry35Ab1 and the aforementioned proteins: 1) No competitive binding to rootworm BBMV was observed for competitor proteins when used in excess with ^125^I-Cry35Ab1 alone or combined with unlabeled Cry34Ab1, and 2) No competitive binding to rootworm BBMV was observed for unlabeled Cry34Ab1 and Cry35Ab1, or a combination of the two, when used in excess with ^125^I-Cry3Aa, or ^125^I-Cry8Ba.

**Conclusions/Significance:**

Combining two or more insecticidal proteins active against the same target pest is one tactic to delay the onset of resistance to either protein. We conclude that Cry34Ab1/Cry35Ab1 are compatible with Cry3Aa, Cry6Aa, or Cry8Ba for deployment as insect resistance management pyramids for in-plant control of western corn rootworm.

## Introduction

Corn rootworms (*Diabrotica* species) are major insect pests of maize in the United States Midwest Corn Belt. Twenty-five years ago annual economic losses were estimated at US $1 billion [1] and since then the acreage, yield, and price of maize have all increased substantially, such that the current economic impact of corn rootworm is likely to be much higher. Corn rootworm larvae feed on the roots of developing corn plants, which impairs water and nutrient uptake, results in corn lodging and reduced harvestability, and ultimately reduces overall crop yield. Three *Diabrotica* species are of primary agronomic importance in the United States. These species are western corn rootworm, *Diabrotica virgifera virgifera* LeConte, northern corn rootworm, *Diabrotica barberi* Smith and Lawrence, and Mexican corn rootworm, *Diabrotica virgifera zeae* [2].

Corn rootworm management practices include chemical insecticides, crop rotation with soybeans and, more recently, in-plant solutions based on *Bacillus thuringiensis* (Bt) crystal (Cry) insecticidal proteins [3], [4], [5], [6], [7]. The ability to effectively control field populations of *Diabrotica* spp. has been challenged by the propensity of *Diabrotica* spp. to develop resistance or change behavior and thereby overcome these crop protection practices. Western corn rootworm has demonstrated the ability to develop resistance to several chemical insecticides [8]. In recent years, females of a soybean adapted population have developed the behavior of laying eggs in soybean fields, thus reducing the effectiveness of crop rotation [9], [10]. As a result, Bt maize expressing insecticidal proteins to control corn rootworm has been rapidly adopted as an alternative technology for control of *Diabrotica* spp. The first Bt maize product for control of corn rootworms, based on Cry3Bb1, was deregulated in 2003 by the US EPA [3]. A recent report from Gassmann et al. [11] describes field-derived populations of western corn rootworm that have lower susceptibility to the Bt protein Cry3Bb1, raising concerns over the possibility of western corn rootworm resistance to Cry3Bb1 resulting from continued use of Cry3Bb1 as the sole corn rootworm resistance trait.

Clearly, more robust approaches to insect resistance management (IRM) are needed to protect the long-term durability of rootworm control technology. For Bt maize, a strategy combining highly effective insect control traits with a non-Bt maize refuge is implemented in the USA as an approach to delay the evolution of resistance to transgenic Bt crops [12], [13]. The refuge strategy requires nearby non-Bt crop refuge where the non-transgenic isoline crop does not produce a Bt insecticidal protein. The refuge provides unselected, susceptible insect populations that survive and mate with potential Bt-resistant individuals from the Bt crop. Refuges are thought to be more effective with increasing levels of insect mortality caused by the Bt crop. To date, documented cases of field-relevant Bt resistance are inherited as a recessive trait (reviewed in [14]) and the heterozygous offspring are susceptible to the Bt maize trait. Thus, the refuge is expected to delay the spread of the resistance alleles in the population.

Another tactic to mitigate the development of Bt-resistant insects is the concept of combining or pyramiding more than one insect resistance gene in the same crop plant. Combining proteins with independent mechanisms of action (MOA) will delay the development of resistant insect populations because only individuals that have acquired resistance to both MOAs will be resistant to crop plants expressing the combined trait genes [15]. Insecticidal proteins compatible for insect resistance management are those with distinct differences in their MOA that therefore have a low probability of cross resistance.

Bt Cry proteins show highly specific activity against certain insect species [16]. Bt Cry proteins intoxicate susceptible larvae by targeting and disrupting the midgut epithelium. The MOA for classical three-domain Cry proteins is well understood. The most widely accepted model for Bt Cry protein mode of action proposes sequential binding to insect midgut epithelial cells followed by pore formation and cell lysis [17], [18]. Mode I insect resistance to Bt Cry proteins is characterized by reduced Cry protein binding, recessive trait inheritance and several hundred fold resistance [19]. In lepidopteran pests, genetic loci for resistance determinants that map to loss of binding have been characterized as mutations in receptors such as cadherins [14], [20], [21], [22] and aminopeptidases [23], [24]. In Coleopteran insects, Cry3Aa has been shown to bind metalloprotease in *Leptinotarsa decemlineata* [25] and cadherin in *Tenebrio molitor* [26], while Cry7Ab3 was recently demonstrated to bind cadherin in *Henosepilachna vigintioctomaculata* [27]. Further, a novel cadherin gene was characterized in *D. virgifera virgifera* [28]. Therefore, insect brush border membrane vesicle (BBMV) binding site interactions that infer different receptors can be used as one criterion to select candidate Bt proteins for combination in IRM pyramids.

Cry34Ab1/Cry35Ab1 proteins have been developed for in-plant control of corn rootworms [5], [29] and are marketed as Herculex® RW traits, or in combination with the Cry1Fa lepidopteran active Bt protein as Herculex® XTRA, in corn hybrids derived from Dow AgroSciences event DAS-59122-7 [4]. Cry34Ab1 and Cry35Ab1 comprise a binary insecticidal protein pair. These proteins are structurally different from classical three-domain Cry proteins such as Cry3Aa [30]. In this report, we provide evidence that Cry34Ab1/Cry35Ab1 do not share western corn rootworm BBMV binding sites with Coleopteran-active Bt proteins Cry3Aa, Cry6Aa [31], [32] or Cry8Ba [33].

## Materials and Methods

### BBMV preparation

Insect midguts were isolated from third instar western corn rootworm larvae. Brush border membrane vesicles (BBMV) were prepared from the isolated midguts using the method of Wolfersberger et al. [34]. Leucine aminopeptidase was used as a marker of membrane proteins during BBMV preparation. Leucine aminopeptidase activity in crude homogenates and BBMV preparations were determined as previously described [35]. The enrichment of Leucine aminopeptidase activity in BBMV preparations ranged from 10 to 15 fold relative to that in crude homogenates. The final BBMV pellet was resuspended in 50% diluted ice cold homogenate buffer (0.3 M Mannitol, 5 mM EGTA, 17 mM Tris–HCl, pH 7.5). Total protein concentration of the BBMV preparation was measured using the method of Bradford [36]. BBMV preparations were snap frozen in liquid nitrogen and stored at −80°C in 100 or 200 µl aliquots until use.

### Full-length Cry protein preparation

Cry protein inclusion bodies produced from recombinant *Pseudomonas fluorescens* clones MR1253 and MR1636 (expressing Cry34Ab1 and Cry35Ab1 proteins, respectively) were resuspended separately in 25 ml of 100 mM sodium citrate buffer, pH 3.0, in a 50-ml conical tube [29]. The tubes were placed on a gently rocking platform at 4°C overnight to extract full-length Cry34Ab1 and Cry35Ab1 proteins. The extracts were centrifuged at 30,000× g for 30 min at 4°C and supernatants containing full-length Cry proteins were retained and stored at 4°C for later use.

Cry3Aa, Cry6Aa, and Cry8Ba protein inclusions from recombinant *P. fluorescens* strains MR832, DPf13032, and DPf159, respectively, were resuspended in 100 mM sodium carbonate buffer, pH 11.0 (for Cry3Aa), or in 50 mM CAPS [3-(cyclohexamino)1-propanesulfonic acid] buffer, pH 10.5 (for Cry6Aa and Cry8Ba). Full-length Cry protoxins were extracted in the basic buffer as described above and stored at 4°C for later use.

### Protease activation of Cry protoxins

Full-length Cry35Ab1, Cry3Aa, and Cry8Ba were digested with chymotrypsin (for Cry35Ab1) or trypsin (for Cry3Aa and Cry8Ba) to generate active protein core fragments [37], [38], [39]. Solubilized full-length Cry35Ab1 was incubated with bovine pancreatic chymotrypsin (Sigma, St. Louis, MO) at 50∶1 (w/w ratio = Cry protein∶enzyme) in 100 mM sodium citrate buffer, pH 3.0, at 4°C with gentle shaking for 2–3 days. Full-length Cry3Aa and Cry8Ba were incubated with bovine pancreatic trypsin (Sigma, St. Louis, MO) at a 20∶1 (w/w ratio = Cry protein∶enzyme) in 100 mM sodium carbonate buffer, pH 11.0 ( Cry3Aa), or 50 mM CAPS buffer, pH 10.5 (Cry8Ba) at room temperature for 1–3 hours. Complete proteolysis was confirmed by SDS-PAGE analysis. The molecular masses of the full-length Cry35Ab1, Cry3Aa, and Cry8Ba are approximately 44, 73, and 134 kDa, and their chymotrypsin-resistant or trypsin-resistant cores were 40, 55, and 57 kDa, respectively. Activated Cry proteins were used for all experiments in this study except for Cry34Ab1 and Cry6Aa which were used as full-length proteins.

### Purification of Cry proteins

Cry35Ab1, Cry3Aa, and Cry8Ba proteolytic polypeptides and full-length Cry34Ab1 and Cry6Aa were purified as follows. Protease digestion reactions were centrifuged at 30,000× g for 30 min at 4°C to remove lipids, and the resulting supernatant was concentrated by 5-fold using an Amicon Ultra regenerated cellulose centrifugal filter device (10,000 Molecular Weight Cutoff; Millipore). The sample buffers were then changed to 20 mM sodium citrate buffer, pH 3.5, for Cry34Ab1 and Cry35Ab1, and to 10 mM CAPS buffer, pH 10.5 for Cry3Aa, Cry6Aa, and Cry8Ba by dialysis at 4°C overnight. The final volumes were adjusted to 15–20 ml using the corresponding buffer for purification using an ATKA Explorer liquid chromatography system (Amersham Biosciences). For Cry35Ab1, buffer A was 20 mM sodium citrate buffer, pH 3.5, and buffer B was 20 mM sodium citrate buffer, pH 3.5, +1 M NaCl. A 5-ml HiTrap SP column (GE Healthcare Life Sciences, GEHL) was used for chromatography. After the column being equilibrated with buffer A, the Cry35Ab1 solution was injected into the column at a flow rate of 5 ml/min. Elution was performed using gradient 0–100% of buffer B at 5 ml/min with 1 ml per fraction. For Cry3Aa, Cry6Aa, and Cry8Ba, buffer A was 10 mM CAPS, pH 10.5, and buffer B was 10 mM CAPS, pH 10.5, +1 M NaCl. A 5-ml Capto Q column (GEHL) was used and the all other procedures were similar to that for Cry35Ab1. Column fractions containing target proteins were visualized on SDS-PAGE gels and pooled. The buffer was changed for the purified Cry35Ab1 chymotrypsin core with 20 mM Bis-Tris, pH 6.0 [29] as described above by dialysis. For the purified Cry3Aa, and Cry8Ba trypsin-resistant cores, and full-length Cry6Aa, salt was removed by dialysis against 10 mM CAPS, pH 10.5 at 4°C overnight. Full-length Cry34Ab1 solubilized in acidic buffer was sufficiently pure for labeling and binding assays. Protein samples were analyzed on SDS-PAGE and quantified on a Typhoon imaging system (GEHL) with BSA as a standard.

### Iodination of Cry proteins

Prior to radio-labeling, non-radioactive iodination was conducted to assess the impact of iodination on Cry protein insecticidal activity. Full-length Cry34Ab1 and Cry6Aa, or protease-activated Cry35Ab1 and Cry3Aa were iodinated using NaI and Pierce® Iodination Beads (Pierce Biotechnology) in multiple 1,200-µl reactions each containing two iodination beads and 2 mM NaI. Optimal buffers were determined to be 100 mM Bis-Tris, pH 6.0 for Cry35Ab1, 100 mM sodium citrate, pH 5.0 for Cry34Ab1, and 100 mM phosphate buffer, pH 8.0 for Cry3Aa and Cry6Aa. The concentration of these Cry proteins in iodination reaction was 0.5 mg/ml, and the reaction time was 3 min with gentle shaking at room temperature for all the Cry proteins except Cry34Ab1. Cry34Ab1 was iodinated for four different time periods: 1, 2, 3, and 4 min to obtain four different levels of iodination. The reactions were stopped by removing the protein solutions from the beads. The iodination reaction buffers were dialyzed against 20 mM sodium citrate, pH 3.5 for both Cry34Ab1 and Cry35Ab1, and against 10 mM CAPS, pH 10.5 for the other proteins. Finally, after concentration using Amicon-10 or 30 MW cutoff tubes, the iodinated Cry proteins were analyzed by SDS-PAGE and quantified with BSA as a standard. Intact mass analysis methodology [40] was utilized to confirm incorporation of iodine into the proteins (data not shown). The iodinated Cry proteins were stored at 4°C for biological activity assays.

### Bioassay

Native or iodinated Cry34Ab1, Cry35Ab1, Cry3Aa, and Cry6Aa were tested in overlay diet feeding assays to determine whether iodination affected biological activity against western corn rootworm neonates. Western corn rootworm eggs were received from Crop Characteristics, Inc. (MN). The bioassays were conducted in 24-well titer plates (BioQuip Products, Inc., CA) for Cry34/35Ab1 and Cry6Aa or 128-well plastic trays (C-D International, NJ) for Cry3Aa. Each well contained 1.5 mL of Dow AgroSciences proprietary corn rootworm diet. A 60–80-µL aliquot of aqueous solubilized proteins or control solution was delivered onto the diet surface of each well as needed to meet desired dose (µg/cm^2^). Four to 8 wells were treated per sample. Combinations of native and iodinated proteins of Cry34Ab1 full length/Cry35Ab1 chymotrypsin-resistant core, Cry6Aa full length, and Cry3Aa trypsin-resistant core were tested at 50/50, 100, and 400 µg/cm^2^, respectively. For controls, 20 mM sodium citrate pH 3.5 and 10 mM CAPS pH 10.5 buffers were used. The treated samples were air dried and then covered with breathable lids (Diversified Biotech, MA, or C-D International). Within 24–48 hours of egg hatching, individual larvae were picked up with a natural hair paintbrush and deposited on the treated diet surface, 2–5 larvae per well. The bioassays were held under controlled environmental conditions (28°C, 40–60% RH, 16∶8 [L∶D] photoperiod). After 5 days, the number of live and dead insects, as well as the pool weights of surviving insects were recorded. The assays were replicated 3–4 times. Data were grouped by toxin and corresponding buffer and analyzed using JMP® Pro 9.0.1 (2010 SAS Institute Inc.). Statistical analyses on the mortality count data and larval weights were performed by contingency analysis (Chi-square Likelihood Ratio test) and analysis of variance (ANOVA) with a means comparison (Tukey Kramer HSD), respectively.

### ^125^I-labeling of Cry proteins

Purified full-length Cry34Ab1 and Cry6Aa, chymotrypsinized Cry35Ab1, and trypsinized Cry3Aa and Cry8Ba, were labeled using ^125^I for specific and competition binding assays with Pierce® Iodination Beads (Pierce Biotechnology) and Na^125^I (PerkinElmer, MA). The labeling was conducted in a 100-µl reaction volume for all these proteins using the similar procedures described for iodination. ^125^I-labeled Cry proteins were separated from free Na^125^I in the solution using a desalting column (Pierce Biotechnology) and kept at 4°C in the labeling buffer. The radioactivity in 1-µl protein solution was measured with a COBRAII Auto-Gamma counter (Perkin Elmer/Packard, Waltham, MA) to estimate specific radioactivity. The specific radioactivity of the iodinated Cry proteins was typically 1–5 µCi/µg.

### Binding assays

Specific binding assays were performed using ^125^I-labeled Cry proteins as described previously [41]. To determine specific binding and estimate the binding affinity (disassociation constant, Kd) and binding site concentration (Bmax) of Cry35Ab1 and the competitor protein Cry3Aa to the insect BBMV, a series of increasing concentrations of either ^125^I-Cry35Ab1 or ^125^I-Cry3Aa were incubated with a given concentration (0.1 mg/ml) of the insect BBMV in 150 µl of 20 mM Bis-Tris, pH 6.0, 100 mM KCl, supplemented with 0.1% BSA at room temperature for 60 min with gentle shaking. Cry protein bound to the BBMV was separated from free protein in the suspension by centrifugation at 12,000× g at room temperature for 8 min. The pellet was washed twice with 900 µl of ice-cold the same buffer containing 0.1% BSA. The radioactivity remaining in the pellet was measured with a COBRAII Auto-Gamma counter (Perkin Elmer/Packard, Waltham, MA) and considered total binding. Another series of binding reactions were set up side-by-side, and a 500–1,000-fold excess of unlabeled corresponding Cry protein was included in each of the binding reactions to fully occupy all specific binding sites on the BBMV; this was a measure of non-specific binding. Specific binding was estimated by subtracting the non-specific binding from the total binding. The Kd and Bmax values of these Cry proteins were estimated using the specific binding against the concentrations of the labeled Cry protein used by running GraphPad Prism 5.01 (GraphPad Software, San Diego, CA). The charts were made using either Microsoft Excel or GraphPad Prism programs. The experiments were replicated at least three times. Specific binding assays for ^125^I-Cry6Aa and ^125^I-Cry8Ba on western corn rootworm BBMV were conducted, and then the two binding parameters (Kd and Bmax) were calculated. Since Cry34Ab1 and Cry35Ab1 function together, the specific binding of ^125^I-Cry35Ab1 was also tested in the presence of Cry34Ab1 at the molar ratio of 50∶1 (Cry34A1∶^125^I-Cry35Ab1). The molar ratio of 50∶1 (Cry34Ab1∶^125^I-Cry35Ab1) was used to ensure the maximum effect of Cry34Ab1 on specific binding of ^125^I-Cry35Ab1. Specific binding assay of ^125^I-Cry34Ab1 was attempted but no specific binding was observed (data not shown).

### Competition binding assays

Competition binding assays were conducted to determine if Cry35Ab1 and competitor proteins Cry3Aa, Cry6Aa, and Cry8Ba share binding sites on western corn rootworm gut membrane. For homologous competition binding assays of Cry35Ab1 and Cry3Aa, increasing amounts (0 to 1,000 nM) of unlabeled Cry35Ab1 or Cry3Aa were first mixed with 5 nM labeled Cry35Ab1 or Cry3Aa, and then incubated with the BBMV at 0.1 mg/ml in 150 µl of 20 mM Bis-Tris, pH 6.0, 100 mM KCl, supplemented with 0.1% BSA at room temperature for 60 min. The percentages of ^125^I-Cry35Ab1 or ^125^I-Cry3Aa bound to the BBMV were determined for each of the reactions as compared to their initial specific binding in the absence of unlabeled competitors. Similarly, homologous competition binding of ^125^I-Cry35Ab1 was assayed in the presence of unlabeled Cry34Ab1 at 250 nM (a molar ratio of 50∶1 = 250-nM Cry34Ab1∶5-nM ^125^I-Cry35Ab1). In addition, homologous competition binding assays for Cry6Aa, and Cry8Ba were also performed in the same buffer (20 mM Bis-Tris, pH 6.0, 100 mM KCl, supplemented with 0.1% BSA) as described above.

Heterologous competition binding assays between ^125^I-Cry3Aa and unlabeled Cry34Ab1, unlabeled Cry35Ab1 alone, or unlabeled Cry35Ab1+Cry34Ab1 were performed to determine if Cry34Ab1 and Cry35Ab1 share binding sites with Cry3Aa. Before we optimized the ratio of Cry34Ab1 and Cry35Ab1 for maximum binding of Cry35Ab1, a 1∶3 molar ratio (Cry35Ab1∶Cry34Ab1) was used to compete with labeled Cry3Aa because a 1∶3 molar ratio is typically used in bioassays to measure mortality of Cry34Ab1/Cry35Ab1. In optimization studies a 1∶20 molar ratio of Cry35Ab1∶Cry34Ab1 resulted in maximum binding of Cry35Ab1. Cry35Ab1 binding did not further increase with further increased amounts of Cry34Ab1 (data not shown). To ensure enough Cry34Ab1 was present in the reaction to facilitate Cry35Ab1 binding a 1∶50 molar ratio of Cry35Ab1/Cry34Ab1 was used in subsequent experiments. The heterologous competition binding assay was conducted with increasing amounts of unlabeled Cry34Ab1, unlabeled Cry35Ab1 alone, or the Cry35Ab1+Cry34Ab1 mixture. The experiments were replicated at least three times. Reciprocal heterologous competition binding assays were also performed using ^125^I-Cry35Ab1 alone or the mixture of 5-nM ^125^I-Cry35Ab1 and 250-nM unlabeled Cry34Ab1 (1∶50 molar ratio) with unlabeled Cry3Aa as a competitor. Similarly, heterologous competition binding assays between Cry35Ab1 alone or the mixture of Cry35Ab1 and Cry34Ab1 at a 1∶50 molar ratio (Cry35Ab1∶Cry34Ab1) with Cry6Aa, or Cry8Ba were performed separately.

## Results and Analyses

### Effect of iodination on Cry proteins

**Figure 1:**
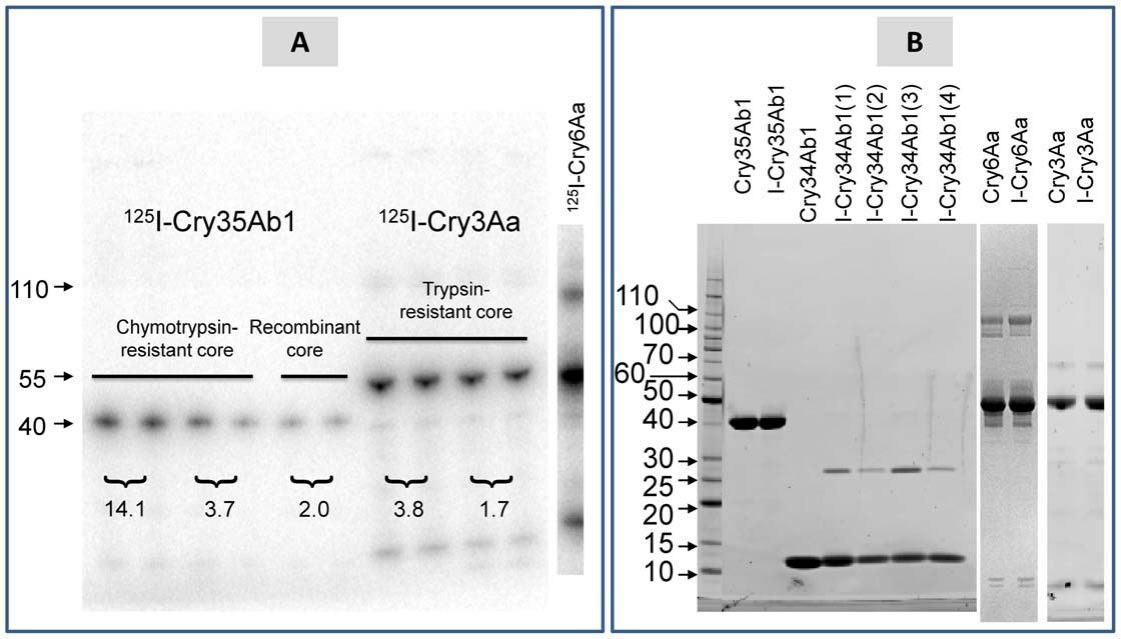
Effect of iodination on stability of Cry proteins. (**A**) Autoradiograph of ^125^I-labeled Cry35Ab1, ^125^I-Cry3Aa and ^125^I-Cry6Aa. Cry35Ab1 chymotrypsin-resistant core was the product of chymotrypsin digestion and Cry35Ab1 recombinant core was expressed in *Pseudomonas fluorescens*. Proteins were labeled with different levels of ^125^I as indicated (µCi/µg protein). (**B**) SDS-PAGE of native and nonradio-iodinated Cry35Ab1, Cry34Ab1, Cry6Aa and Cry3Aa. Cry and I-Cry represent native and iodinated forms of Cry proteins. Cry34Ab1 was iodinated for 1, 2, 3, or 4 min, all of which showed lack of activity in diet bioassays on western corn rootworm larvae.

The effect of iodination on Cry protein biological activity was ascertained (Table 1). On SDS-PAGE gels iodinated Cry35Ab1 migrated as a single band relatively free of contaminant proteins (Fig. 1A and 1B) and iodination did not affect biological activity of Cry35Ab1. However, iodination of Cry34Ab1 drastically reduced activity of the binary insecticidal protein in the presence of native or iodinated Cry35Ab1. Interestingly, a proportion of iodinated Cry34Ab1 was in the form of dimers with a molecular size of approximately 28 kDa (Fig. 1B). Dimers of approximately 108 kDa were observed in both native and iodinated preparations of Cry6Aa (Fig. 1B). Cry6Aa monomers and dimers were able to be radio-iodinated as indicated in Fig. 1A. Mortality of iodinated Cry6Aa treatment was only slightly reduced and larval weight was similar to native Cry6Aa. Iodinated Cry3Aa migrated as a single band relatively free of contaminant proteins. Mortality for iodinated Cry3Aa treatment was significantly reduced but biological activity was not completely abolished as indicated by the reduction in larval weights for iodinated Cry3Aa.

**Table 1:** Mean percent mortality and larval weight (± SE) of the *D. virgifera virgifera* when exposed to native and iodinated Cry34Ab1/35Ab1, Cry6Aa and Cry3Aa for 5 days on overlay diet bioassay.

Cry toxins^a^	Concentration (µg/cm^2^)	% Mortality^b^	Larval weight (mg)^b^
Cry34Ab1/35Ab1	50/50	91.0±3.7^A^	0.02±0.01^D^
Cry34Ab1^i^/35 Ab1	50/50	34.7±5.0^B^	0.19±0.02^C^
Cry34Ab1/35Ab1^i^	50/50	89.4±2.8^A^	0.04±0.02^D^
Cry34AB1^i^/35Ab1^i^	50/50	11.5±3.8^C^	0.28±0.03^B^
20 mM Na citrate pH 3.5 buffer	0	16.4±5.5^C^	0.42±0.01^A^
Cry6Aa	100	97.5±1.4^A^	0.003±0.003^B^
Cry6Aa ^i^	100	87.0±1.4^B^	0.06±0.02^B^
10 mM CAPS pH 10.5 buffer	0	3.9±1.3^C^	0.42±0.03^A^
Cry3Aa1	400	35.9±16.6^A^	0.13±0.01^C^
Cry3Aa1^i^	400	4.5±2.2^B^	0.41±0.02^B^
10 mM CAPS pH 10.5 buffer	0	2.1±2.1^B^	0.71±0.06^A^

a Cry protein followed by the letter (^i^) denotes that the protein was iodinated. Unlabelled Cry proteins were in native form. Cry34/35Ab1 was comprised of full length Cry34Ab1 and Cry35Ab1 chymotrypsin-resistant core, whereas full length Cry6Aa and Cry3Aa1 trypsin-resistant core were used.

b For each Cry protein, means followed by the same letter within the column are not significantly different at *Pr* = 0.05. For statistical analysis data were grouped by Cry protein and corresponding buffer. Statistical analysis on the mortality count data was performed using Chi-square Likelihood Ratio test. Analysis of variance (ANOVA) with means comparison was conducted using Tukey-Kramer HSD test on larval weight data (4 replications, n = 20). For Cry3A, similar statistical analyses were used except there were 3 replications, n = 16.

Native, unlabelled Cry34Ab1 binds to distinct BBMV proteins on ligand blots (data not shown). However, because Cry34Ab1 was inactivated by iodination we were unable to measure ^125^I-Cry34Ab1 specific binding to western corn rootworm BBMV. Further attempts to detect Cry34Ab1 specific binding using proteins labeled with fluorescein-5-maleimide via engineered cysteine residues at the Cry34Ab1 N-terminus or C-terminus were also unsuccessful.

### Specific binding of Cry35Ab1

**Figure 2:**
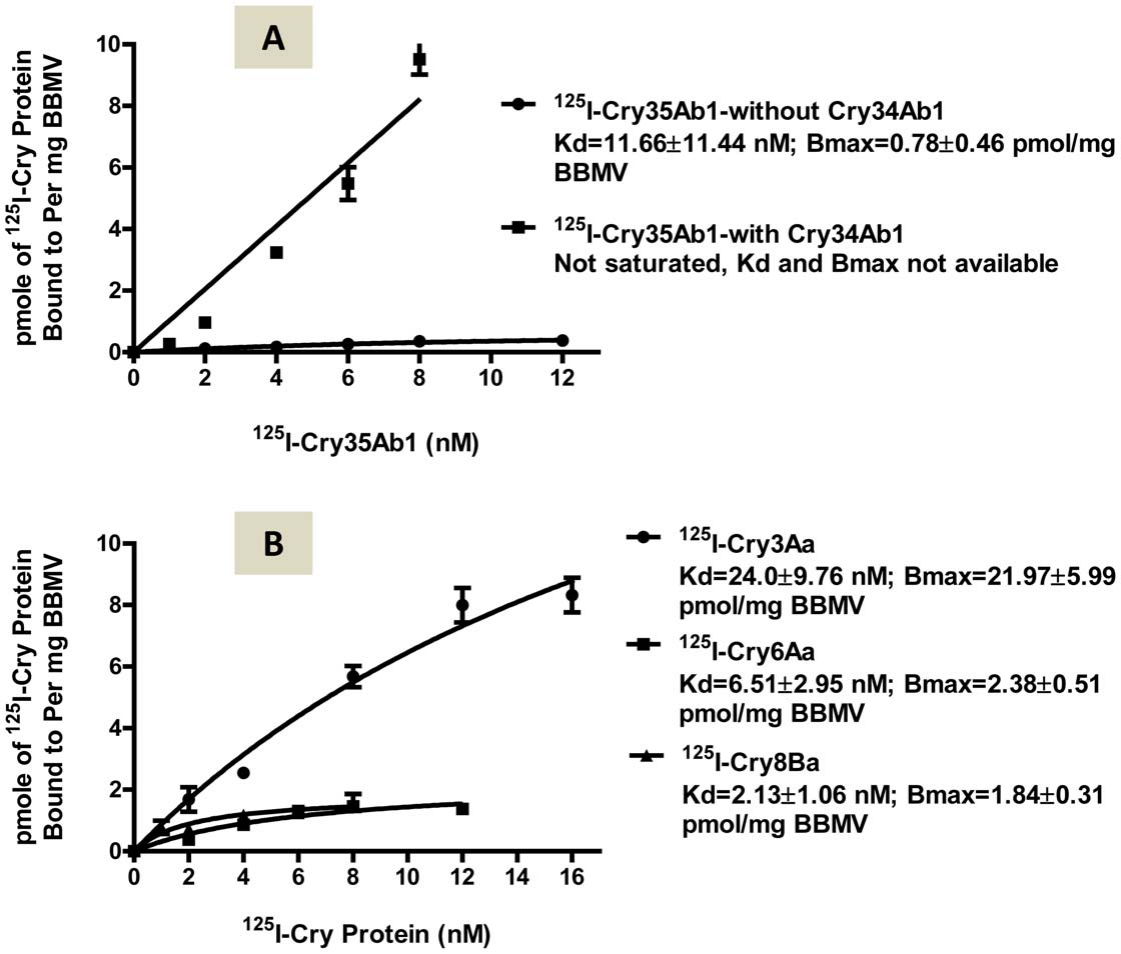
Specific binding of ^125^I-labeled Cry35Ab1, Cry3Aa, Cry6Aa and Cry8Ba. (**A**) Specific binding of ^125^I-Cry35Ab1 (chymotrypsin-resistant core) alone or in the presence of Cry34Ab1 (1∶50 molar ratio of ^125^I-Cry35Ab1:Cry34Ab1 across all concentrations tested), and (**B**) Specific binding of ^125^I-Cry3Aa (trypsin-resistant core), ^125^I-Cry6Aa (full length), and ^125^I-Cry8Ba (trypsin-resistant core) as a function of input radiolabeled Cry proteins to BBMV at 0.1 mg/ml.

In either the presence or absence of unlabeled Cry34Ab1 (1∶50 molar ratio = ^125^I-Cry35Ab1∶Cry34Ab1), radio-labeled Cry35Ab1 exhibited specific binding to western corn rootworm BBMV (Fig. 2A). In the absence of Cry34Ab1, the dissociation constant (Kd) of ^125^I-Cry35Ab1 was 11.66±11.44 nM, and the Bmax was 0.78±0.46 pmol/mg BBMV, indicating a low level of specific binding. However, the binding of ^125^I-Cry35Ab1 increased over 10-fold in the presence of Cry34Ab1, indicating that Cry34Ab1 facilitated binding of Cry35Ab1. In the presence of Cry34Ab1, the specific binding of ^125^I-Cry35Ab1 accounted for most of the total binding of ^125^I-Cry35Ab1, i.e., non-specific binding was relatively low by comparison to Cry35Ab1 alone (data not shown). In this case the Kd and Bmax values could not be calculated because specific binding was not saturated when Cry34Ab1 was included in the binding reaction. These data indicate that Cry34Ab1 facilitates the binding of Cry35Ab1 to western corn rootworm BBMV and the specific binding of Cry35Ab1 alone may be not significant when compared to that in the presence of Cry34Ab1.

### Specific binding of Cry3Aa, Cry6Aa, and Cry8Ba

^125^I-Cry3Aa showed specific binding to western corn rootworm BBMV, with a dissociation constant (Kd) of 24.0±9.76 and binding site concentrations (Bmax) of 21.97±5.99 (Fig. 2B). These Kd and Bmax values could be slightly different if the specific binding were fully saturated. The specific binding of full-length Cry6Aa was calculated to have a Kd value of 6.51±2.95 nM and a Bmax value of 2.38±0.51 pmol/mg BBMV (Fig. 2B). Both the binding affinity and binding site concentration are higher than those of Cry35Ab1 alone. Specific binding of Cry8Ba was detected as well and the binding affinity was higher than all other Cry proteins tested with a Kd = 2.13±1.06 nM, but the binding capacity was 1.84±0.31 pmol/mg BBMV, a moderate level among the proteins tested (Fig. 2B).

### Competition binding between Cry34Ab1/Cry35Ab1 and Cry3Aa, Cry6Aa, or Cry8Ba

**Figure 3:**
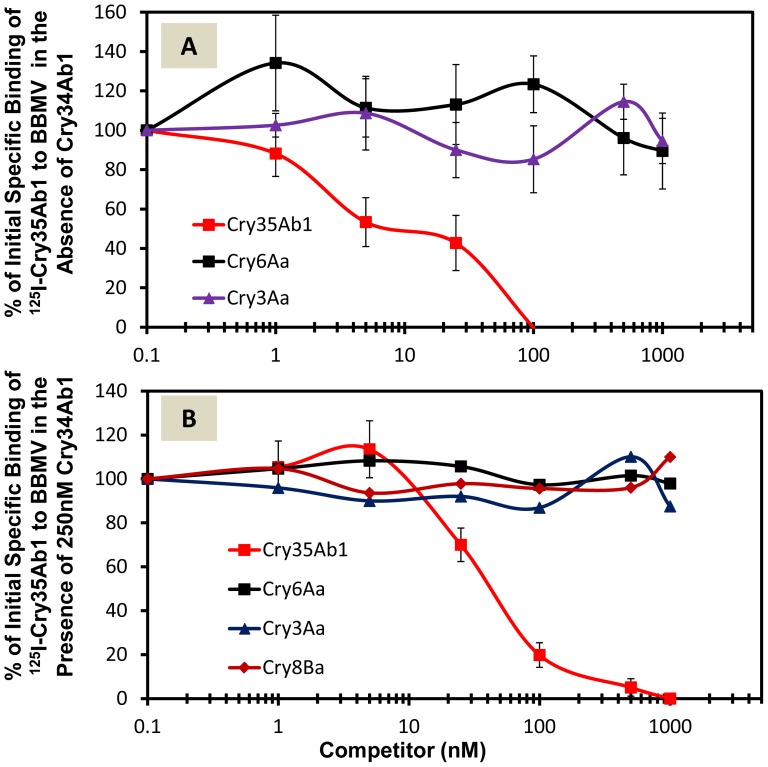
Cry34Ab1/Cry35Ab1 competition binding with Cry proteins. (**A**) Competition binding between ^125^I-Cry35Ab1 in the absence of Cry34Ab1 and unlabeled Cry35Ab1, Cry3Aa, or Cry6Aa. (**B**) Competition binding between ^125^I-Cry35Ab1 in the presence of 250 nM Cry34Ab1 and unlabeled Cry35Ab1, Cry3Aa, Cry6Aa or Cry8Ba. The data for homologous competition between labeled and unlabeled Cry35Ab1 in panel B were an average of 9 replicates (3 replicates for 3 independent experiments); all other data were the average of triplicate assays. The concentration of western corn rootworm BBMV was 0.1 mg/ml. The concentration of ^125^I-Cry protein was 5 nM, and the increasing concentrations of the unlabeled competitors were indicated. Error bars represent standard deviation.

**Figure 4:**
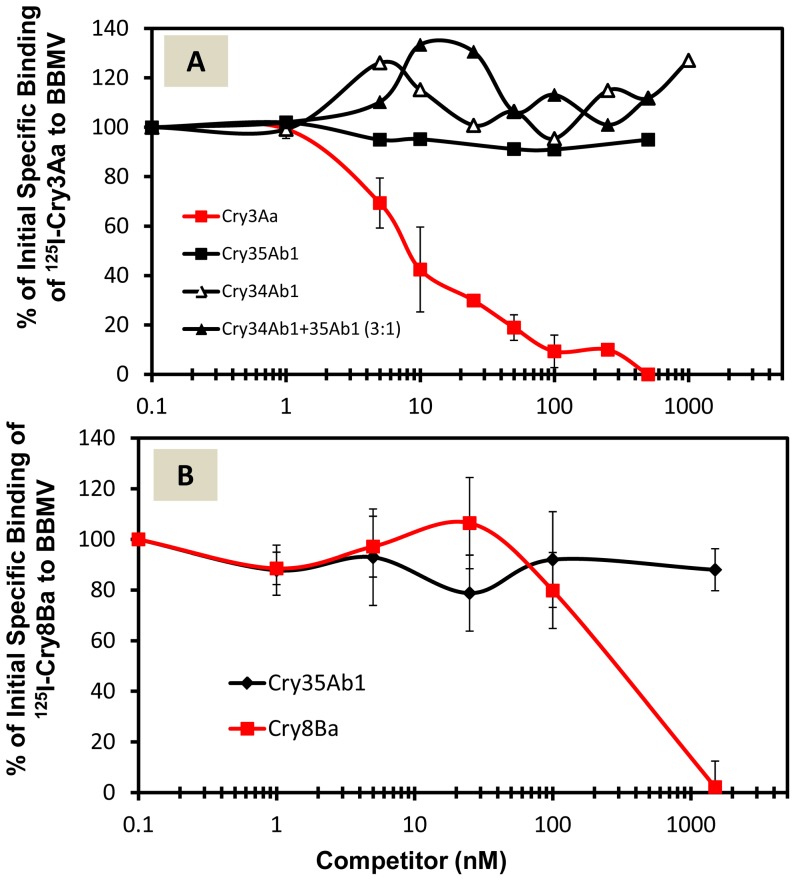
Competition binding between unlabeled Cry34Ab1/35Ab1 and ^125^I-labeled other Cry proteins. (**A**) for ^125^I-Cry3Aa and (**B**) for ^125^I-Cry8Ba. The concentration of western corn rootworm BBMV was 0.1 mg/ml. The concentration of ^125^I-Cry protein was 5 nM. Increasing concentrations of the unlabeled competitors are indicated. Error bars represent standard deviation.

Homologous competition of ^125^I-Cry35Ab1 was demonstrated in the absence or presence of Cry34Ab1. Cry3Aa and Cry6Aa did not compete for ^125^I-Cry35Ab1 binding in the absence (Fig. 3A) or presence (Fig. 3B) of unlabeled Cry34Ab1. Cry8Ba did not compete for ^125^I-Cry35Ab1 binding in the presence of unlabeled Cry34Ab1 (Fig. 3B). In a reciprocal competition binding experiment, we observed homologous competition when the molar concentration of Cry3Aa was increased to 5 nM (equal to ^125^I-Cry3Aa) (Fig. 4A). However, neither Cry35Ab1 nor Cry34Ab1 alone nor the mixture of Cry35Ab1+Cry34Ab1 (1∶3 molar ratio), competed for ^125^I-Cry3Aa binding (Fig. 4A). Similarly, we observed homologous competition between ^125^I-Cry8Ba and unlabeled Cry8Ba when the concentration of unlabeled Cry8Ba was increased to 100 nM (equivalent to 20 fold excess of ^125^I-Cry8Ba). However, unlabeled Cry35Ab1 did not compete for ^125^I-Cry8Ba binding (Fig. 4B). These data suggest that Cry34Ab1, Cry35Ab1, and their mixture (Cry34Ab1/Cry35Ab1) do not share binding sites with Cry3Aa, Cry6Aa or Cry8Ba.

## Discussion and Conclusions

Sustainable in-plant control of *Diabrotica* spp. depends on innovation and robust IRM plans to prevent or delay the development of resistant insect populations. In the United States, refuges are required for Bt maize [12], which are expected to be more effective when planted along with Bt maize that causes high levels of insect mortality. This approach to IRM is known as the “high dose/refuge’ strategy. Pyramided Bt traits are expected to dramatically delay the development of resistant insect populations and provide a theoretical basis for reduced refuge size [15]. Bt proteins active against corn rootworms, e.g. Cry34Ab1/Cry35Ab1 [5], [29], mCry3Aa [7], Cry3Bb1 [6] and several other candidate Cry proteins, e.g. Cry6Aa [31], [32], Cry8Ba [33], along with new technology such as RNAi [42] provide options to develop corn varieties with pyramided insect resistance traits.

The concept of pyramiding Bt proteins to mitigate the development of resistant insect populations requires that the proteins do not share a common high-level resistance mechanism in the insect pest. Mode 1 resistance to Cry proteins, the most common type of insect resistance to Bts, is characterized by recessive inheritance and reduced Cry protein binding to midgut receptors [14], [19]. Determination of interactions on insect midgut membranes is one measure of whether two proteins share a common binding site, and are therefore compatible when combined as pyramided insect resistance traits.

The present study focused on the assessment of the potential for receptor mediated cross resistance by measuring binding site interactions on western corn rootworm midgut BBMV. For the binary insecticidal proteins Cry34Ab1/Cry35Ab1 we successfully radiolabeled Cry35Ab1 by iodination as a reagent for BBMV binding studies. However, iodination of Cry34Ab1 abolished the biological activity of this protein and specific binding of ^125^I-Cry34Ab1 to BBMVs was not detectable even though native Cry34Ab1 binds distinct BBMV protein bands on ligand blots (data not shown).

As a first step toward understanding Cry34Ab1/Cry35Ab1 binding interactions we demonstrated ^125^I-Cry35Ab1 specific binding to sites on western corn rootworm BBMV. Interestingly, unlabeled Cry34Ab1 facilitated Cry35Ab1 binding in what seems to be a cooperative manner (Figure 2A). This observation is consistent with the need for both proteins to attain full membrane permeabilization and insecticidal activity [29], [37]. Cry34Ab1/Cry35Ab1 cooperative binding is reminiscent of a report showing enhanced membrane binding between the *B. spharaericus* binary toxin components BinA and BinB on *Anopheles gambiae* brush border membrane fragments [43]. Mechanistically, enhanced Cry35Ab1 binding in the presence of unlabeled Cry34Ab1 might reflect Cry35Ab1 binding to a Cry34Ab1/BBMV complex or, alternatively, binding of a Cry34Ab1/Cry35Ab1 complex to specific BBMV sites.

Our next objective was to determine binding site interactions between Cry34Ab1/Cry35Ab1 and other corn rootworm-active proteins in assays using ^125^I-Cry35Ab1. Two lines of evidence presented here support the lack of shared binding sites for Cry34Ab1/Cry35Ab1 and Cry3Aa, Cry6Aa or Cry8Ba: 1) No competitive binding to rootworm BBMV was observed for competitor proteins when used in excess with ^125^I-Cry35Ab1 alone or combined with unlabeled Cry34Ab1, and 2) No competitive binding to rootworm BBMV was observed for unlabeled Cry34Ab1 and Cry35Ab1, or a combination of the two, when used in excess with ^125^I-Cry3Aa or ^125^I-Cry8Ba. These results indicate that midgut receptors involved in the mechanism of action differ between Cry34Ab1/Cry35Ab1 and the other proteins, and therefore suggest a low likelihood of receptor-mediated cross resistance between Cry34Ab1/Cry35Ab1 and the other Cry proteins examined in this study.

From both functional and structural perspectives Cry34Ab1/Cry35Ab1 are unique among the known Cry proteins active on western corn rootworm. In addition to the membrane interactions presented here, the primary sequence and three dimensional crystal structures of Cry34Ab1 and Cry35Ab1 differ from the other proteins tested [44], [45], [46]. Therefore, we conclude that Cry34Ab1/Cry35Ab1 are compatible with one or more insect resistance proteins selected from Cry3Aa, Cry6Aa or Cry8Ba for new IRM pyramids for in-plant control of western corn rootworm. Consistent with this conclusion, Gassmann et al. (2011) recently demonstrated that field-derived western corn rootworm populations with reduced susceptibility to Cry3Bb1 corn, showed unchanged susceptibility to Cry34Ab1/Cry35Ab1 proteins [11].

In summary, this study provides the first evidence for Cry34Ab1/Cry35Ab1 specific binding sites on western corn rootworm BBMV, enhanced specific binding of Cry35Ab1 by Cry34Ab1, and that Cry34Ab1/Cry35Ab1 binding sites are independent from binding sites for Cry3Aa, Cry6Aa, and Cry8Ba. These data along with the unique protein structures of Cry34Ab1/Cry35Ab1 suggest a different mechanism of action for Cry34Ab1/Cry35Ab1 that enables several possibilities for new Bt combinations for pyramided traits targeting western corn rootworm.

## References

[1] Metcalf RL (1986) Foreword, pp. vii–xv, In Krysan JL, Miller TA [eds.], Methods for the study of pest *Diabrotica*. Springer-Verlag, NY.

[2] Drees BM, Levine E, Stewart JW, Sutter GR, Tollefson JJ (1999) Corn rootworms. Page 66 in Handbook of Corn Insects, Steffey KL et al., eds., Entomological Society of America, Lanham, Md.

[3] United States Environmental Protection Agency (2003) *Bacillus thuringiensis* Cry3Bb1 protein and the genetic material necessary for its production (vector zmir13l) in event MON863 corn fact sheet. EPA Publ. No.730-F-03-01.

[4] United States Environmental Protection Agency website. Current & Previously Registered Section 3 PIP Registrations. Available: http://www.epa.gov/oppbppd1/biopesticides/pips/pip_list.htm. Accessed 2012 Dec 4.

[5] Moellenbeck DJ, Peters ML, Bing JW, Rouse JR, Higgins LS, et al (2001) Insecticidal proteins from *Bacillus thuringiensis* protect corn from corn rootworms. Nat Biotechnol 19: 668–72. 11433280 10.1038/90282

[6] Siegfried BD, Vaughn TT, Spencer T (2005) Baseline susceptibility of western corn rootworm (Coleoptera: Crysomelidae) to Cry3Bb1 *Bacillus thuringiensis* toxin. J Econ Entomol 98: 1320–4. 16156586 10.1603/0022-0493-98.4.1320

[7] Walters FS, Stacy CM, Lee MK, Palekar N, Chen JS (2008) An engineered chymotrypsin/cathepsin G site in domain I renders *Bacillus thuringiensis* Cry3A active against Western corn rootworm larvae. Appl Environ Microbiol 74: 367–74. 18024675 10.1128/AEM.02165-07 PMC2223250

[8] Wright RJ, Scharf ME, Meinke LJ, Zhou XG, Siegfried BD, et al (2000) Larval susceptibility of an insecticide-resistant western corn rootworm (Coleoptera: Chrysomelidae) population to soil insecticides: laboratory bioassays, assays of detoxification enzymes, and field performance. J Econ Entomol 93: 7–13. 14658504 10.1603/0022-0493-93.1.7

[9] Gray ME, Sappington TW, Miller NJ, Moeser J, Bohn MO (2009) Adaptation and invasiveness of western corn rootworm: intensifying research on a worsening pest. Annu Rev of Entomol 54: 303–321. 19067634 10.1146/annurev.ento.54.110807.090434

[10] O'Neil ME, Gray ME, Smyth CA (1999) Population characteristics of a western corn rootworm (Coleoptera: Chrysomelidae) strain in east central Illinois corn and soybean fields. J Econ Entomol 92: 1301–1310.

[11] Gassmann AJ, Petzold-Maxwell JL, Keweshan RS, Dunbar MW (2011) Field-evolved resistance to Bt maize by Western corn rootworm. PLoS One 6 7: e22629. 21829470 10.1371/journal.pone.0022629 PMC3146474

[12] United States Environmental Protection Agency (1998) The Environmental Protection Agency's White Paper on Bt Plant-Pesticide Resistance Management. Washington, D.C.: Environmental Protection Agency.

[13] United Stated Environmental Protection Agency (2001) Biopesticides Registration Action Document: Bacillus thuringiensis (Bt) Plant Incorporated Protectants. Washington, D.C.: Environmental Protection Agency, Office of Pesticide Programs, Biopesticides, and Pollution Prevention Division.

[14] Heckel DG, Gahan LJ, Baxter SW, Zhao JZ, Shelton AM, et al (2007) The diversity of Bt resistance genes in species of Lepidoptera. J Invertebr Pathol 95: 192–7. 17482643 10.1016/j.jip.2007.03.008

[15] Roush RT (1998) Two-toxin strategies for management of insecticidal transgenic crops: can pyramiding succeed where pesticide mixtures have not? Philos Trans R Soc Lond B Biol Sci 353: 1777–1786.

[16] van Frankenhuyzen K (2009) Insecticidal activity of *Bacillus thuringiensis* crystal proteins. J Invertebr Pathol 101: 1–16. 19269294 10.1016/j.jip.2009.02.009

[17] Schnepf E, Crickmore N, Van Rie J, Lereclus D, Baum J, et al (1998) *Bacillus thuringiensis* and its pesticidal crystal proteins. Microbiol Mol Biol Rev 62: 775–806. 9729609 10.1128/mmbr.62.3.775-806.1998 PMC98934

[18] Bravo A, Gill SS, Soberon M (2007) Mode of action of *Bacillus thuringiensis* Cry and Cyt toxins and their potential for insect control. Toxicon 49: 423–35. 17198720 10.1016/j.toxicon.2006.11.022 PMC1857359

[19] Tabashnik BE, Liu Y, Malvar T, Heckel DG, Masson L, et al (1998) Insect resistance to *Bacillus thuringiensis*: uniform or diverse? Phil Trans R Soc Lond B 353: 1751–1756.

[20] Gahan LJ, Gould F, Heckel DG (2001) Identification of a gene associated with Bt resistance in *Heliothis virescens* . Science 293: 857–860. 11486086 10.1126/science.1060949

[21] Yang Y, Chen H, Wu Y, Yang Y, Wu S (2007) Mutated cadherin alleles of *Helicoverpa armigera* conferring resistance to *Bacillus thuringiensis* toxin Cry1Ac detected in the field. Appl Environ Microbiol 73: 6939–6944. 17827322 10.1128/AEM.01703-07 PMC2074965

[22] Zhao J, Jin L, Yang Y, Wu Y (2010) Diverse cadherin mutations conferring resistance to *Bacillus thuringiensis* toxin Cry1Ac in *Helicoverpa armigera* . Insect Biochem Mol Biol 40: 113–118. 20079435 10.1016/j.ibmb.2010.01.001

[23] Herrero S, Gechev T, Bakker PL, Moar WJ, de Maagd RA (2005) *Bacillus thuringiensis* Cry1Ca-resistant *Spodoptera exigua* lacks expression of one of four aminopeptidase N genes. BMC Genomics 6: 96. 15978131 10.1186/1471-2164-6-96 PMC1184072

[24] Rajagopal R, Sivakumar S, Agrawal N, Malhotra P, Bhatnagar RK (2002) Silencing of midgut aminopeptidase N of *Spodoptera litura* by double-stranded RNA establishes its role as *Bacillus thuringiensis* toxin receptor. J Biol Chem 277: 46849–51. 12377776 10.1074/jbc.C200523200

[25] Ochoa-Campuzano C, Real MD, Martínez-Ramírez AC, Bravo A, Rausell C (2007) An ADAM metalloprotease is a Cry3Aa *Bacillus thuringiensis* toxin receptor. Biochem Biophys Res Commun 362: 437–442. 17714689 10.1016/j.bbrc.2007.07.197

[26] Fabrick J, Oppert C, Lorenzen MD, Morris K, Oppert B, et al (2009) A novel *Tenebrio molitor* cadherin is a functional receptor for *Bacillus thuringiensis* Cry3Aa toxin. J Biol Chem 284: 18401–18410. 19416969 10.1074/jbc.M109.001651 PMC2709378

[27] Song P, Wang Q, Nangong Z, Su J, Ge D (2012) Identification of *Henosepilachna vigintioctomaculata* (Coleoptera: Coccinellidae) midgut putative receptor *for Bacillus thuringiensis* insecticidal Cry7Ab3 toxin. J Invertebr Pathol 109: 318–22. 22306353 10.1016/j.jip.2012.01.009

[28] Sayed A, Nekl ER, Siqueira HA, Wang HC, ffrench-Constant RH, et al (2007) A novel cadherin-like gene from western corn rootworm, *Diabrotica virgifera virgifera* (Coleoptera: Chrysomelidae), larval midgut tissue. Insect Mol Biol 16: 591–600. 17725800 10.1111/j.1365-2583.2007.00755.x

[29] Ellis RT, Stockhoff BA, Stamp L, Schnepf HE, Schwab GE, et al (2002) Novel *Bacillus thuringiensis* binary insecticidal crystal proteins active on western corn rootworm, *Diabrotica virgifera virgifera* LeConte. Appl Environ Microbiol 68: 1137–1145. 11872461 10.1128/AEM.68.3.1137-1145.2002 PMC123759

[30] Li JD, Carroll J, Ellar DJ (1991) Crystal structure of insecticidal delta-endotoxin from *Bacillus thuringiensis* at 2.5 A resolution. Nature 353: 815–21. 1658659 10.1038/353815a0

[31] Narva KE, Schwab GE, Bradfisch GA (1993) Novel *Bacillus thuringiensis* gene encoding coleopteran-active toxin. US patent 5,186,934.

[32] Thompson M, Knuth M, Cardineau G (1999) *Bacillus thuringiensis* toxins with improved activity. US patent 5,874,288.

[33] Michaels TE, Narva KE, Foncerrada L (1996) *Bacillus thuringiensis* toxins active against scarab pests. US patent 5,554,534.

[34] Wolfersberger MG, Luthy P, Maurer A, Parenti P, Sacchi F, et al (1987) Preparation and partial characterization of amino acid transporting brush border membrane vesicles from the larval midgut of the cabbage butterfly (*Pieris brassicae*). Comp Biochem Physiol 86A: 301–308.

[35] Li H, Oppert B, Higgin RA, Huang F, Zhu KY, et al (2004) Comparative analysis of proteinase activities of *Bacillus thuringiensis*-resistant and -susceptible *Ostrinia nubilalis* (Lepidoptera: Crambidae). Insect Biochem Mol Biol 34: 753–762. 15262280 10.1016/j.ibmb.2004.03.010

[36] Bradford MM (1976) A rapid and sensitive method for the quantitation of microgram quantities of protein utilizing the principle of protein–dye binding. Anal Biochem 72: 248–254. 942051 10.1016/0003-2697(76)90527-3

[37] Masson L, Schwab G, Mazza A, Brousseau R, Potvin L, et al (2004) A novel *Bacillus thuringiensis* (PS149B1) containing a Cry34Ab1/Cry35Ab1 binary toxin specific for western corn rootworm *Diabrotica virgifera virgifera* LeConte forms ion channels in lipid membranes. Biochemistry 43: 12349–57. 15379574 10.1021/bi048946z

[38] Rausell C, García-Robles I, Sánchez J, Muñoz-Garay C, Martínez-Ramírez AC, et al (2004) Role of toxin activation on binding and pore formation activity of the *Bacillus thuringiensis* Cry3 toxins in membranes of *Leptinotarsa decemlineata* (Say). Biochim Biophys Acta 1660: 99–105. 14757225 10.1016/j.bbamem.2003.11.004

[39] Carroll J, Li J, Ellar DJ (1989) Proteolytic processing of a coleopteran-specific δ-endotoxin produced by *Bacillus thuringiensis* var. *tenebrionis* . Biochem J 261: 99–105. 2549968 10.1042/bj2610099 PMC1138787

[40] Domon B, Aebersold R (2006) Mass spectrometry and protein analysis. Science 312 5771: 212–217. 16614208 10.1126/science.1124619

[41] Li H, Oppert B, Gonzalez–Cabrera J, Ferré J, Higgins RA, et al (2004) Binding analysis of Cry1Ab and Cry1Ac with membrane vesicles from *Bacillus thuringiensis*–resistant and –susceptible *Ostrinia nubilalis* (Lepidoptera: Crambidae). Biochem Biophys Res Commun 323: 52–57. 15351700 10.1016/j.bbrc.2004.08.054

[42] Baum JA, Bogaert T, Clinton W, Heck GR, Feldmann P, et al (2007) Control of coleopteran insect pests through RNA interference. Nat Biotechnol 25: 1322–1326. 17982443 10.1038/nbt1359

[43] Charles JF, Silva-Filha MH, Nielsen-LeRoux C, Humphreys MJ, Berry C (1997) Binding of the 51- and 42-kDa individual components from the *Bacillus sphaericus* crystal toxin to mosquito larval midgut membranes from *Culex* and *Anopheles* sp. (Diptera: Culicidae). FEMS Microbiol Lett 156: 153–159. 9368375 10.1111/j.1574-6968.1997.tb12721.x

[44] Narva KE, Schnepf HE, Knuth M, Pollard MR, Cardineau G, et al. (2002) Pesticidal proteins. U.S. patent 6,372,480.

[45] Schnepf HE, Narva KE, Evans SL (2007) Rationally designed variants of the Cry35 δ-endotoxin for use in the control of Coleoptera. US patent 7309785.

[46] Schnepf HE (2009) Modified Cry34 proteins and methods of controlling plant pests, including rootworms, with these modified proteins. US patent 7524810.

